# Mechanical Properties of *Boehmeria nivea* Natural Fabric Reinforced Epoxy Matrix Composite Prepared by Vacuum-Assisted Resin Infusion Molding

**DOI:** 10.3390/polym12061311

**Published:** 2020-06-09

**Authors:** Fabio da Costa Garcia Filho, Fernanda Santos da Luz, Lucio Fabio Cassiano Nascimento, Kestur Gundappa Satyanarayana, Jaroslaw Wieslaw Drelich, Sergio Neves Monteiro

**Affiliations:** 1 Military Institute of Engineering—IME, Materials Science Program, Praça General Tibúrcio 80, Urca, Rio de Janeiro 22290-270, Brazil; fsl.santos@gmail.com (F.S.d.L.); lucio_coppe@yahoo.com.br (L.F.C.N.); snevesmonteiro@gmail.com (S.N.M.); 2 Poornaprajna Scientific Research Institute (PPISR) Sy. No. 167, Poornaprajnapura Bidalur Post, Devanahalli, Bangalore 562 110, India; gundsat42@hotmail.com; 3 Department of Materials Science and Engineering, Michigan Technological University, Houghton, MI 49931, USA; jwdrelich@mtu.edu

**Keywords:** *Boehmeria nivea* fabric, natural fiber/epoxy composites, mechanical behavior, VARIM technique

## Abstract

Natural lignocellulosic fibers and corresponding fabrics have been gaining notoriety in recent decades as reinforcement options for polymer matrices associated with industrially applied composites. These natural fibers and fabrics exhibit competitive properties when compared with some synthetics such as glass fiber. In particular, the use of fabrics made from natural fibers might be considered a more efficient alternative, since they provide multidirectional reinforcement and allow the introduction of a larger volume fraction of fibers in the composite. In this context, it is important to understand the mechanical performance of natural fabric composites as a basic condition to ensure efficient engineering applications. Therefore, it is also important to recognize that ramie fiber exhibiting superior strength can be woven into fabric, but is the least investigated as reinforcement in strong, tough polymers to obtain tougher polymeric composites. Accordingly, this paper presents the preparation of epoxy composite containing 30 vol.% *Boehmeria nivea* fabric by vacuum-assisted resin infusion molding technique and mechanical behavior characterization of the prepared composite. Obtained results are explained based on the fractography studies of tested samples.

## 1. Introduction

The sustainable development of our society could be associated with the application of natural-based materials for engineering applications. However, the reliable application of these materials in favor to current used materials awakens new technological and environmental challenges. The case of natural fibers is iconical. For many centuries, natural fibers were used as raw material for manufacturing simple items like clothes, baskets, and house cover, among others. However, since the end of the 20th century, they emerged as a valuable option for the substitution of synthetic fibers for the reinforcement of polymer composites, as disclosed in numerous review articles [1,2,3,4,5,6,7,8,9,10,11,12]. In fact, some select natural fibers were reported to display tensile strengths above 1000 MPa [13], which is higher than most structural steels [14], although the former show larger scatter in the values. In spite of the above-mentioned properties of NLFs, the heterogeneity exhibited by these fibers is one of the major challenges to the wider application of these materials. The obtained properties of NLFs may be defined as a function of the fiber diameter and composition, but even the quality of the soil or the season in which the fiber is harvested will impact on it. Nevertheless, these natural fibers are considered viable alternatives to replace glass fiber for the reinforcement of polymeric materials, owing to superior specific strength [15,16,17]. Moreover, several innovative studies showed that the use of natural fibers for composite reinforcement is suitable for relevant engineering applications, such as automobile parts, cyclist helmets, building construction, and even bulletproof personal vests [18,19,20,21,22,23,24,25].

Relevant reasons justify the raising interest for natural lignocellulosic fibers (NLFs), as they offer societal, economic, environmental and technical benefits when compared to synthetic fibers [13]. In particular, the environmental benefits are associated with sustainable aspects such as renewability, biodegradability, recyclability, CO_2_ neutrality and reduced carbon footprint. These advantages contribute to reducing fossil fuel-based energy consumption and corresponding climate change. Other advantages are their low cost, as well as the fact of being widely grown around the world, which allows the possibility of their cultivation as source of income to many communities [26]. In spite of these advantages, the use of NLFs presents some drawbacks. Their application as reinforcement of polymeric materials depends on adhesion to the matrix. The adhesion between two dissimilar phases usually can be considered in four structural levels: molecular, micro, meso and macro level. However, the concept of interface is commonly considered as regarding to the first two. At molecular level chemical structures is defined by the formation of Van der Waals forces, acid-base interactions and chemical bonds between the two phases. As for the micro level, bond strength and interfacial shear stress are the properties used to characterize the load transfer through the interface [27]. Indeed, studies have shown that the adhesion in molecular level of NLFs to the most commonly used polymer matrices is generally not satisfactory, which compromises the other levels of structural adhesion of the composite. This is due to the hydrophilic nature of the NLFs in contrast to the hydrophobic character of the polymer matrices [1]. The moisture absorbed by the fibers acts as a separating agent between the fibers and the matrix. Nevertheless, several physical and chemical treatments as well as production techniques have been proposed to enhance the interfacial adhesion between NLF reinforcement and polymeric matrix [28,29,30,31]. Jacob et al. [28] showed that due to the presence of hydroxyl groups, chemical treatments that activate cellulose molecules in the fiber could modify surface characteristics such as: adhesion, wetting, porosity or surface tension. Alkaline, anhydride and silanation modification treatments are some of the popular surface treatments for NLFs. More recently, graphene oxide modification has emerged as the most efficient treatment for surface properties enhancement [25,29]. Gholampour et al. [30] discussed several physical treatments that could enhance the interfacial adhesion between NLFs and polymeric matrix such as: plasma, ultraviolet (UV) exposure and heat treatment.

Of the NLFs, the *Boehmeria nivea*, known as ramie fiber, is among those exhibiting superior tensile strength. The *Boehmeria nivea* fiber originated from China, where it is commonly named as “Chinese grass” [32]. Besides China, which is the main producer, other countries such as Brazil, Indonesia, India and Cuba also contribute to the production of over 120,000 kg/year [33]. *Boehmeria nivea* is an herbaceous perennial plant from the Urticaceae family, which due to its unique characteristics is used as raw material for natural textile [34]. In fact, the capability to be woven could be considered as a major advantage when used as reinforcement for polymer matrix composites. Indeed, it provides multidirectional reinforcement and greater volume fraction of reinforcement material could be achieved. These natural fibers and fabric exhibit competitive properties when compared with some synthetics like the glass fiber. In particular, the use of fabrics made from natural fibers might be considered a more efficient alternative, since they provide multidirectional reinforcement and allow the introduction of a larger volume fraction of fibers in the composite. In this context, it is important to understand the mechanical performance of natural fabric composites as a basic condition to ensure efficient engineering applications. Recognizing that studies of various lignocellulosic fibers and their fabrics have been reported [34,35,36,37,38,39,40,41,42], the *Boehmeria nivea* fiber exhibiting superior strength can be woven into fabric, but is the least investigated as reinforcement in strong, tough polymers to obtain tougher polymeric composites. Accordingly, this paper presents the preparation of epoxy composite containing 30 vol.% of *Boehmeria nivea* fabric via the vacuum-assisted resin infusion molding (VARIM) technique and mechanical behavior characterization of the prepared composite. Obtained results are explained based on the fractograpy studies of tested samples.

## 2. Experimental Procedure

**Figure 1:**
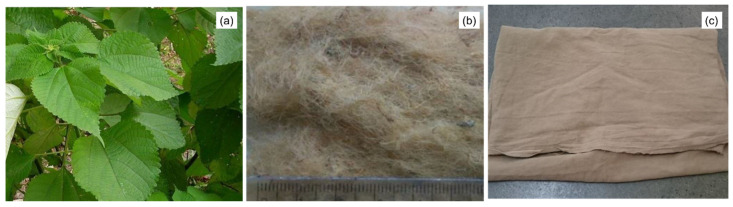
*Boehmeria nivea* (**a**) plant, (**b**) fiber bundle and (**c**) fabric.

Figure 1 shows macrophotographs of *Boehmeria nivea* (a) plant, (b) fibers and (c) fabric. The fabric used in this study, seen in Figure 1c showing its general aspects, was supplied by Rose Natural Healthy Items Wholesale, China.

**Figure 2:**
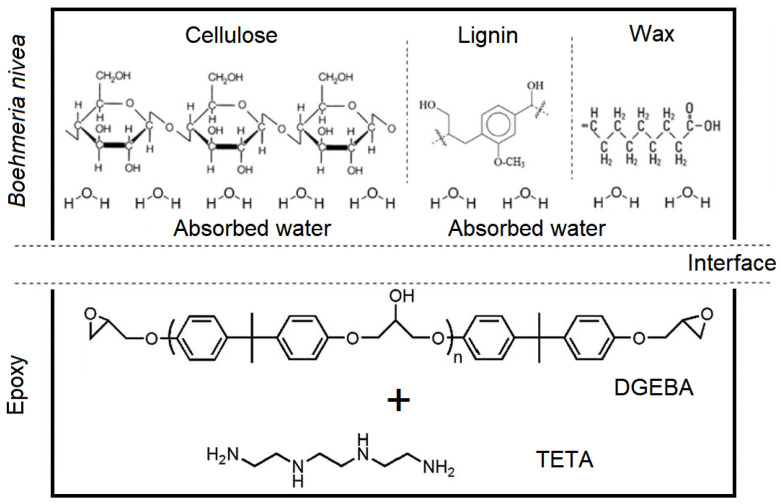
Schematic model of the molecular structure of Boehmeria nivea fabric/epoxy matrix interface.

The polymer used as matrix was a commercially available epoxy resin—bisphenol A diglycidyl ether (DGEBA) resin mixed with triethylenetetramine (TETA) hardener, stoichiometric phr = 13, both supplied by EpoxyFiber, Brazil. The volumetric fraction of fabric reinforcement was set as 30% in order to allow a comparison to other NLFs/epoxy composites [35,36,37,38,39,40,41]. Figure 2 schematically illustrates a suggested model for the molecular structure of the fabric facing an epoxy macromolecule in the matrix. Interaction at the fiber/matrix interface could be mostly accomplished by either Van der Waals forces or hydrogen bonding.

However, prior to the composite production the *Boehmeria nivea* fabric was cut into the mold format and cleaned in an ultrasonic bath with deionizing water for 20 min. Afterwards, the fabrics were placed in an oven at 80 °C for 24 h in order to dry and also reduce the inherent moisture content of the fiber. This is an important step towards improving the interfacial shear strength between the polymeric matrix and the natural fabric reinforcement by reducing the absorbed water favoring the chemical bonding of the composite [30,31].

**Figure 3:**
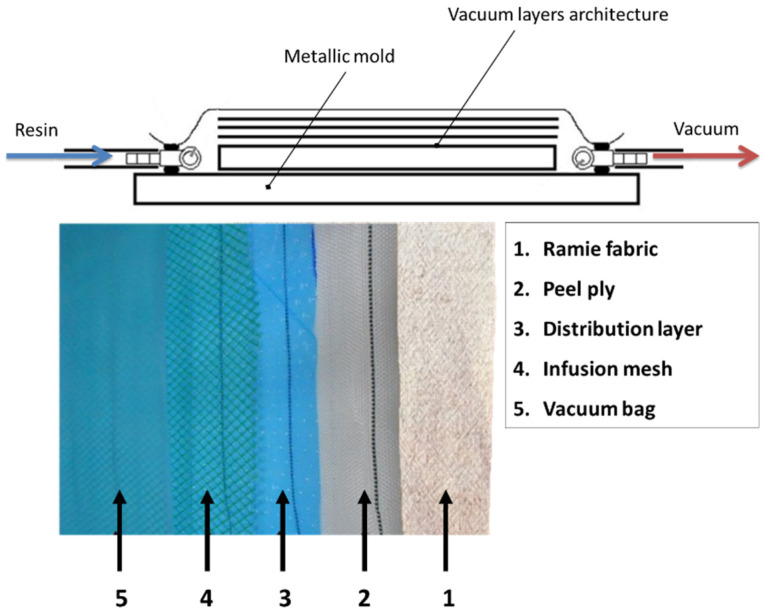
Vacuum-assisted resin infusion molding schematic illustration and layer architecture of the present work.

The composite preparation was made via the vacuum-assisted resin infusion molding (VARIM) technique. Figure 3 schematically illustrates the VARIM process and shows the layered architecture displacement of the produced composite. A simple mechanical pump with maximum capacity of 760 mmHg was used to vacuum the system. Composite specimens, named as ERC, were cured at room temperature for 24 h. Additional neat epoxy specimens were also produced as reference samples.

To assess the mechanical properties of ERC composites, tensile, flexural and impact resistance tests were performed. Tensile specimens with dimensions 250 × 25 × 2.5 mm^3^ were produced and tested as per ASTM D3039 [42]. The analyses of flexural samples with dimensions of 122 × 25 × 10 mm^3^ were made according to ASTM D790 [43]. The final shape of the specimens was obtained by cutting the composite plates in proper dimensions using a band saw, Makita brand. Both tensile tests and three-point bend tests were carried out in a model 5582 Instron machine, with load cell of 1 kN, strain rate of 10^−2^ s^−1^, at room temperature. The response of the material under tensile load also allows other properties such as toughness, ductility or modulus of resilience to be calculated by Equations (1)–(3).

(1) $$\mathrm{Ductility}\left(\%\right)=\left(\frac{l_{1}-l_{o}}{l_{0}}\right)\times 100$$

(2) $$\;\mathrm{Modulus}\;\mathrm{of}\;\mathrm{resilience}\;=\int_{0}^{\varepsilon_{y}}\sigma d\varepsilon \;$$

(3) $$\;\mathrm{Toughness}\;=\int_{0}^{\varepsilon_{f}}\sigma d\varepsilon \;$$

where *l*_0_ is the original length before any load is applied; *l*_1_ is the instantaneous length; ε_y_ is the strain at the yield and ε_f_ is the strain at fracture. Impact tests were conducted in a pendulum EMIC machine, model AIC, using Charpy’s and Izod’s configuration, in accordance with the ASTM D6110 [44] and ASTM D256 [45] standards, respectively. The notched specimens’ dimensions were 122 × 12.7 × 10 mm^3^ for the Charpy’s and 61 × 12.7 × 10 mm^3^ Izod’s impact. For each investigated condition, 6 specimens were tested to ensure statistical validation.

The microstructural characterization of the specimens was performed with the assistance of scanning electron microscopy (SEM) in a model Quanta FEG 250, FEI microscope operating with secondary electrons in the range of 10–20 kV. Image J software was used for further image processing.

## 3. Results and Discussion

### 3.1. Ramie Fabric Characterization

**Figure 4:**
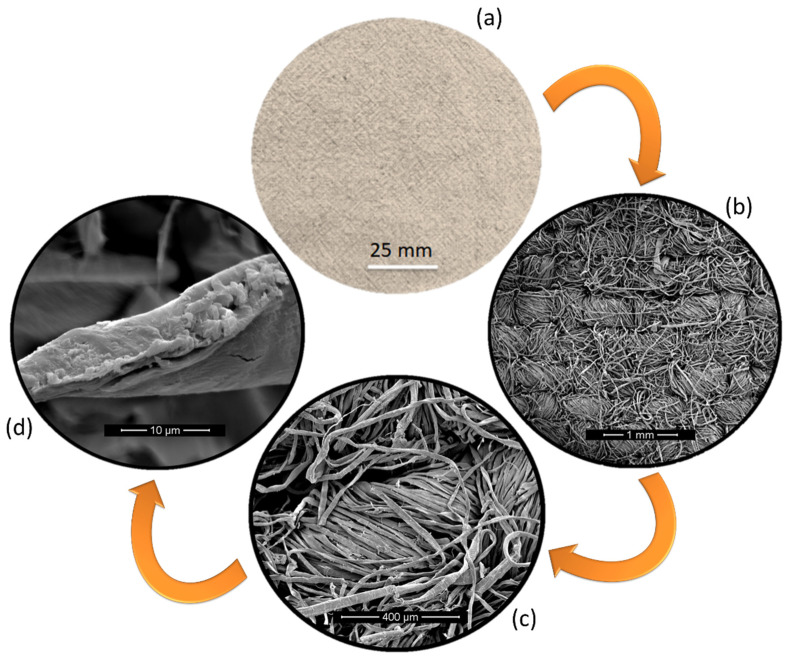
*Boehmeria nivea* from macro to micro (**a**) fabric, (**b**) plain weave, (**c**) yarn, and (**d**) fiber.

Figure 4 shows the aspect of the *Boehmeria nivea* fabric from macro- to microscopic aspects.

In Figure 4a, one may notice the superficial appearance with characteristic color and texture of the *Boehmeria nivea* fabric. The surface area of the fabric was calculated in accordance to the ASTM D3776 standard [46] to be 0.025 g/cm^2^. In Figure 4b it is possible to verify the plain weave of the fabric, which is considered the simplest type of weave. Diameter of the fiber yarn was measured to be 387 ± 59 μm. In Figure 4c, a closer look in a single yarn allows us to notice that it is formed of several fibers with an average diameter of 19 ± 6 μm. Moreover, the fibers are arranged in a helical way, 19.2°. Ma et al. [47] studied the effect of the yarn structure of ramie fiber in their mechanical properties. They showed that the highest tensile strength of a single yarn is obtained for the linear density of 67.3 tex which is associated with a surface twist angle of approximately 20°. This suggests that the structure of the fabric would favor the mechanical properties of the composites. Figure 4d presents the SEM of a single fractured *Boehmeria nivea* fiber. One can verify that unlike other NLFs such as jute, sisal or curaua, the ramie fiber does not display a circular-like shape. Instead, an elliptical-like shape is observed, with b/a ratio of about 2. This kind of shape was also observed in less common NLFs, such as guaruman fiber [48].

### 3.2. Tensile Properties

**Figure 5:**
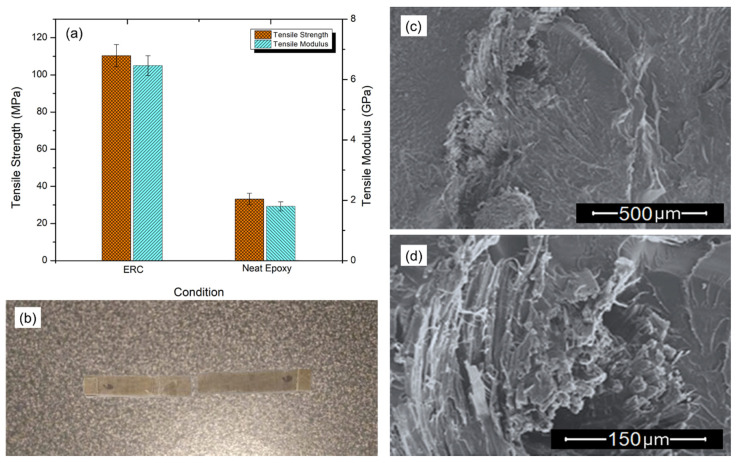
Tensile properties (**a**) results, (**b**) macroscopic failure, (**c**) matrix failure, and (**d**) fiber failure.

Figure 5a shows plots the tensile strength and tensile (Young’s) modulus of the prepared composite (ERC), and those of neat epoxy condition. It is possible to see that the tensile strength of the composite reached over ~110 MPa against about ~30 MPa for the neat epoxy condition. In addition, the tensile modulus also followed similar trend with an increase of over three times for the ERC in comparison to the neat epoxy.

In fact, the significant improvement in the tensile strength could be explained on the basis of the simple fiber–matrix interaction and fracture behavior for polymer matrix reinforced fiber composites. In this unidirectional tensile test, the fibers in the fabric that are aligned with the applied stress will be mainly responsible for absorbing the load that the material has been subjected to. These fibers will act as obstacles for the crack propagation through the epoxy matrix resulting in the enhanced tensile strength. Figure 5b–d show the macrophotograph of a fractured specimen as well as SEM images of the fractured surface. One can see that all specimens’ fractures tend to occur transversally to the tensile axis. As can be seen from Figure 5b, no evidence of fabric participation was observed, which indicates the absence of the pull-out effect. This behavior could also be attributed to the fact that the fibers parallel to the tensile stress, present in the outer face of the specimen during the test, tend to be pulled out from the matrix. Whereas those in perpendicular position tend to hold into the matrix, avoiding the pull-out effect. However, one can see from Figure 5d that the fabric slightly pulls out from the matrix. This is due to a small delamination effect, common in composites reinforced with textile materials, such as natural fabrics. In spite of this effect, the fabric still maintains a reasonable adhesion to the matrix. It can also be noticed that, based on the fracture marks of the epoxy matrix surface, shown in Figure 5c, the fabric acted as a barrier to crack propagation, justifying the higher composite tensile strength as compared to the correspondent value of plain epoxy. These results are similar to those reported by Gu et al. [49], where ramie/epoxy composites were prepared with a slightly higher amount of fabric. They measured a tensile strength of about ~100 MPa for a composite with a fiber mass fraction of 42.5%.

Table 1 presents other properties that could be calculated from the tensile test result, such as toughness, modulus of resilience and ductility. In particular, the toughness and modulus of resilience of the ERC display almost twice of the value calculated for the neat epoxy. On the other hand, ductility was observed to be the same for both materials within the standard deviation.

### 3.3. Flexural Properties

**Figure 6:**
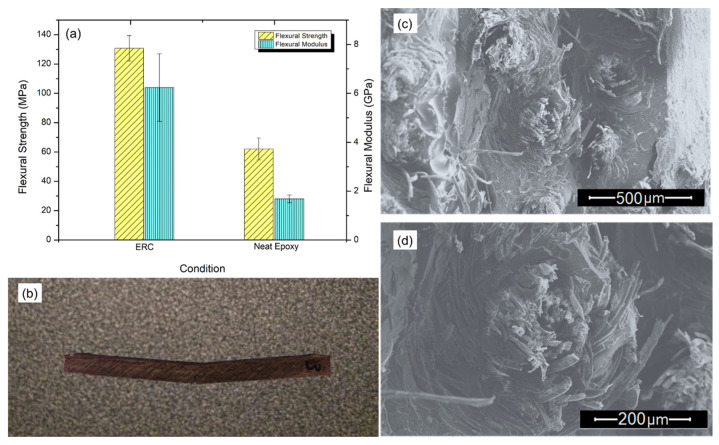
Flexural properties (**a**) results, (**b**) macroscopic failure, (**c**) deflected crack propagation and (**d**) yarn brakeage.

Figure 6a shows the results for flexural strength and flexural modulus for both prepared ERC composite and neat epoxy. Indeed, the *Boehmeria nivea* fabric reinforcement in the epoxy matrix increased significantly both flexural strength and modulus, from about ~60 to over ~130 MPa and around ~2 to almost ~6.5 GPa, respectively.

Figure 6b shows the partial rupture of a specimen after the bend test. A closer look at the macrophotograph suggests that during the fracture of the composite sample, adhesion between the matrix and both the fibers and their fabric has not been lost. The absence of macroscopic evidence of the pull-out of fibers is indicative of an effective adhesion between the fabric reinforcement and the epoxy matrix, which might be associated with the superior flexural resistance achieved by the ERC composite. Figure 6c,d exhibit the fracture surface of the composite after bend test. Figure 6c reveals that the fabric causes the deviation of cracks during their propagation. It is also noticed that the fibers fracture occurs at the same level of the matrix, revealing that the fabric was not pulled out from the epoxy matrix. This behavior was also observed in the tensile strength specimens and, as aforementioned, indicates that the fabric worked as an efficient barrier to crack propagation. In Figure 6d, one again may observe a slight pull-out effect. The strong interaction between the fabric fibers and the epoxy matrix can be related to both the physical treatment of fabric before producing the composite and to the VARIM technique. This is a relevant improvement regarding the commonly weak interface interaction displayed by natural fibers when reinforcing polymeric matrices [1,2,3,4,5,6,7,8,9,10,11,12].

### 3.4. Impact Resistance

**Figure 7:**
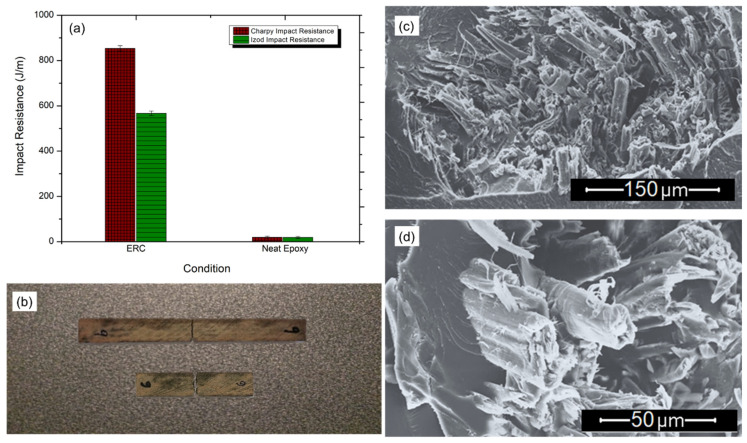
Impact resistance (**a**) results, (**b**) macroscopic failure of Charpy’s and Izod’s specimens, (**c**) several fibers fractured and (**d**) fiber pull out.

Figure 7a presents the impact resistance results for the investigated conditions. From the figure, one can see substantial increase in the impact strength of the composite compared to that of the matrix, underlining the effect of reinforcement by the fabric. The neat epoxy condition is capable of absorbing about ~20 J/m for Charpy or Izod impact configurations, while the ERC was capable of absorbing about ~850 J/m for Charpy and around ~550 J/m for Izod. This large enhancement of impact strength of the composite over that of the matrix could be associated with the ability of the fabric to block the crack propagation in the composite, which demands a greater amount of energy before the composite is fractured.

Figure 7b illustrates the macroscopic aspect of an almost transversal fracture surface with total rupture between the matrix and the fabric for both impact configuration. The complete rupture of the composite reveals that there is no significant slip of the fabric out of the matrix. In Figure 7c, one may notice that the fabric rupture, as in the tensile test, occurs at the same level of the fracture surface, evidencing that fiber slipping did not occur. Figure 7d shows evidence of fiber rupture and pull-out that leads to significant energy absorption, increasing the impact strength. The impact test result helps to justify the superior ballistic performance observed by Monteiro et al. [50] for an epoxy composite reinforced with 30 vol.% of ramie fabric applied in multilayered armor. The amount of 30 vol.% of fabric in the composite was the minimum to guarantee the physical integrity of a second layer plate in the MAS, with observed results similar to those reported for Kevlar^TM^. In all three distinct mechanical tests, it was observed, as aforementioned, that the fabric fibers have not slipped out from the matrix, as generally happens in polymer matrix composites reinforced with non-woven natural fibers.

### 3.5. Comparison with Other NLFs Composites

With a view to understand the importance of the present study, a comparison of the three mechanical properties evaluated for the ERC composite is made with earlier reported values for various epoxy-based composites containing the fabrics of different fibers [35,36,37,38,39,40,41]. Table 2 shows this comparison.

It can be seen from the Table 2 that all the three mechanical properties obtained in the present investigation for the ERC composite are the highest compared to those reported so far. Considering bidirectional fabric, the tensile and flexural strength of the flax/epoxy composites are comparable to those of ERC, but higher than those observed for fique/epoxy and jute/epoxy composites. Furthermore, comparing epoxy composites reinforced with the unidirectional and long fibers of hemp and mallow fibers as well as ramie/polyester composites, one can see that the results of mallow/epoxy and ERC are almost similar. However, the ramie/polyester exhibited higher flexural strength and Charpy impact resistance. This could be associated with the effective participation of the reinforcement in the transfer of load from the matrix—i.e., the composite design. For the unidirectional design, all fibers are aligned in the same direction of the stress; therefore, all of them will be directly loaded. Regarding the bidirectional configuration, half of the fibers are in the same direction of the applied stress and the other half are orthogonal to it. Therefore, while considering the same volumetric amount of reinforcement, the influence of the fiber will be superior in the unidirectional configuration, but the bidirectional design is closer to the desired value for “real” engineering applications, where the loading is usually associated with more than one direction [51].

## 4. Conclusions

The mechanical properties of epoxy composites reinforced with 30 vol.% of *Boehmeria nivea* fabric (ERC) produced by the VARIM technique were investigated and compared to other NLFs/polymer matrix composites.

- Tensile tested composites exhibit a significant increase in both the strength and tensile modulus. Ductility is almost not affected by the introduction of fabric into the epoxy matrix, but other tensile properties, such as modulus of resilience and toughness, also displayed a significant increase in comparison to the neat epoxy.
- A flexural strength of over 130 MPa and a higher flexural modulus (over 6 GPa) were observed for the ERC, corresponding to more than two times those for the neat epoxy reference condition.
- The general improvement found in the impact resistance contributes to justifying the effectiveness of the use of ERC composites for ballistic protection applications. Charpy and Izod impact resistances of ~850 and ~550 J/m were obtained.
- Comparison of three mechanical properties of prepared composite (ERC) with those of epoxy resin containing other NLFs revealed that the properties obtained in the present study are the best and highest reported so far for epoxy resin containing various NLFs. Furthermore, the above also suggests that, to achieve superior mechanical properties, it would be better to have the load transfer from the matrix to the reinforcing fibers in the fabric.
- Fractography studies of the ERC composites carried out using scanning electron microscopy suggested that pre-processing of the fabric and the VARIM technique enhanced the adhesion between the natural fiber fabric and the polymeric matrix.

## Figures and Tables

**Table 1:** Other calculated tensile properties.

Properties	Neat Epoxy	ERC
Toughness	87.8 ± 14.5 MJ/m^3^	184.7 ± 16.8 MJ/m^3^
Modulus of Resilience	12.5 ± 1.1 MJ/m^3^	21.5 ± 5.7 MJ/m^3^
Ductility	3.18 ± 0.31%EL [in 57 mm]	4.71 ± 1.26%EL [in 57 mm]
Elongation at fracture	0.81 ± 0.023 mm	0.88 ± 0.015 mm

**Table 2:** Comparison of mechanical properties of several NLFs reinforcing polymer matrix composites.

Composite (0.3NLFs/0.7polymer)	Fiber Design/Manufacture Method	Tensile Strength (MPa)	Flexural Strength (MPa)	Impact Resistance (J/m)	Reference
ERC	Bidirectional fabric/VARIM	110 ± 6	131 ± 9	^(C)^ 854 ± 12 ^(I)^ 567 ± 10	*PW
Fique/epoxy	Bidirectional fabric/Press molding	47	X	^(C)^ 480 ± 180 ^(I)^ 222 ± 50	35
Jute/epoxy	Bidirectional fabric/Hand lay-up	90	34	^(I)^ 426	36
Flax/epoxy	Bidirectional fabric/Hand lay-up	118	131	X	37
Hemp/epoxy	Unidirectional and long/Press molding	50 ± 4	77 ± 6	X	38
Mallow/epoxy	Unidirectional and long/Press molding	178 ± 18	191 ± 24	^(C)^ 905 ± 95 ^(I)^ 499 ± 35	39, 40
Ramie/polyester	Unidirectional and long/Press molding	89 ± 9	212 ± 12	^(C)^ 1000 ^(I)^ 594	41

*PW = present work; ^(C)^ Charpy impact; ^(I)^ Izod impact.
